# The Possible Role of Mycotoxins in the Pathogenesis of Endometrial Cancer

**DOI:** 10.3390/toxins16060236

**Published:** 2024-05-23

**Authors:** Márkó Unicsovics, Zsófia Molnár, Miklós Mézes, Katalin Posta, György Nagyéri, Szabolcs Várbíró, Nándor Ács, Levente Sára, Zsuzsanna Szőke

**Affiliations:** 1 Department of Obstetrics and Gynecology, Semmelweis University, 1088 Budapest, Hungary; varbiroszabolcs@gmail.com (S.V.); acsnandor@gmail.com (N.Á.); saralevente@gmail.com (L.S.); 2 Department of Animal Biotechnology, Agribiotechnology and Precision Breeding for Food Security National Laboratory, Institute of Genetics and Biotechnology, Hungarian University of Agriculture and Life Sciences, 2100 Gödöllő, Hungary; molnar.zsofia@uni-mate.hu (Z.M.); nagyeri.gyorgy@uni-mate.hu (G.N.); ferenczine.szoke.zsuzsanna@uni-mate.hu (Z.S.); 3 Department of Feed Safety, Institute of Physiology and Nutrition, Hungarian University of Agriculture and Life Sciences, 2100 Gödöllő, Hungary; mezes.miklos@uni-mate.hu; 4 Department of Microbiology and Applied Biotechnology, Institute of Genetics and Biotechnology, Hungarian University of Agriculture and Life Sciences, 2100 Gödöllő, Hungary; posta.katalin@uni-mate.hu; 5 Department of Obstetrics and Gynecology, University of Szeged, 6725 Szeged, Hungary

**Keywords:** endometrial cancer (EC), mycotoxins: aflatoxins (Afs), zearalenone (ZEN), fumonisins, deoxynivalenol (DON), T2/HT2 toxins, endometrium tissue

## Abstract

Endometrial cancer is one of the most common cancer types among women. Many factors can contribute to the development of this disease, including environmental factors and, thus, eating habits. Our study aims to determine the levels of various mycotoxins and their metabolites in the blood serum and endometrial tissue samples of participants with previously proven endometrial cancer and to find possible contributions to cancer development. In the cohort clinical trial, 52 participants aged between 44 and 86 were studied. The participants were divided into two groups: patients or matched controls. All patients had previously histologically diagnosed endometrial cancer. The cancer patients were divided into low-grade endometrioid and low- plus high-grade endometrioid groups. Controls had no history of endometrial malignancy or premalignancy. Blood serum and endometrial tissue samples were obtained from all study patients. We compared the concentrations of total Aflatoxins (Afs), Deoxynivalenol (DON), Ochratoxin-A (OTA), T2-toxin and HT2 toxin (T2/HT2 toxin), Zearalenone (ZEN), alpha-Zearalenol (α-ZOL), and Fumonisin B1 (FB1) in the serum and endometrium between the different study groups. As a result, we can see a significant correlation between the higher levels of Afs and zearalenone and the presence of endometrial cancer. In the case of Afs, DON, OTA, T2/HT2 toxins, ZEN, and alpha-ZOL, we measured higher endometrial concentrations than in serum. Considering the effect of mycotoxins and eating habits on cancer development, our results might lead to further research exploring the relationship between certain mycotoxins and endometrium cancer.

## 1. Introduction

Mycotoxins are secondary metabolites produced by certain filamentous fungi, of which most well-known species belong to the genera *Aspergillus*, *Penicillium*, and *Fusarium*. The conditions required for fungal development and mycotoxin production depend greatly on the substrate on which the fungal species grow. Moreover, climatic conditions lead to significant differences in the occurrence of mycotoxins in human food, influenced by the geographical location and the year of study [1]. Due to their diversity of origin, mycotoxins differ in structure, leading to significant differences in their physicochemical properties and biological effects. Consequently, the toxic effects of mycotoxins vary greatly depending on the compound under study and the species [2,3]. Mycotoxins induce harmful biological effects, e.g., carcinogenic, mutagenic, teratogenic, estrogenic, immunotoxic, nephrotoxic, and neurotoxic effects. The human ingestion of mycotoxins originates from consuming mycotoxin-contaminated plant-based foods and the residues and metabolites in animal products. The greatest human health concern related to mycotoxins is direct cancer risk based on long-term, low-level exposures to carcinogenic toxins, such as aflatoxins (Afs), ochratoxin A (OTA), zearalenone (ZEN), and fumonisins [4,5]. However, the mycotoxin intake in humans is unpredictable due to different diets, such as vegetarian or non-vegetarian, Mediterranean, etc., and the lack of continuous mycotoxin screening of foodstuffs. The most common mycotoxins in our environment and food products are also classified as carcinogenic agents that can play a role in the development of various cancers with sufficient or limited evidence [6]. 

Endometrial cancer cases follow an increasing incidence worldwide and are not only the most common gynecological cancer but the sixth most frequently occurring cancer among women (WCRF). Endometrial malignancies are classified into subtypes based on the novel histological and molecular diagnostics methods. The most common histological type is endometrioid endometrial carcinoma, which is responsible for 80–90% of all cases; however, in terms of progression, estrogen dependency, and therapy resistance, those can be distinguished between low-grade and high-grade types. Four further subgroups can be distinguished based on molecular diagnostics [7]. The first showed somatic inactivating mutations in the exonuclease domain of polymerase epsilon (POLE), the second had a loss of mismatch repair (D-MMR), the third showed a low mutation rate but frequent TP53 mutations, and the fourth had a non-specified molecular profile. Despite increasingly detailed and rich knowledge, the etiology of endometrial carcinoma is still undefined [8]. The development of endometrial cancer has been found to be impacted by the PI3K-PTEN-AKT-mTOR, RAS-MEK-ERK, and WNTβ-catenin signaling pathways. Exposure to aflatoxins, zearalenone, and ochratoxin-A can also have an impact on these signaling pathways [9,10,11,12].

The literature review and analysis showed the possible carcinogenic effects of mycotoxins. The most frequently affected cascades described in the in vitro studies showed a large overlap with the processes that are overexpressed in endometrial cancer [13,14,15]. Based on these results, the question about the possible pathological role of mycotoxins strongly arises. Apart from some previous studies, which suggested the role of ZEN in the development of endometrial carcinoma, until now, there has been a lack of clinical studies revealing the relationship between mycotoxins and endometrial cancer [16].

The purpose of the present study was to obtain a cross-sectional view of mycotoxins that can be associated with low- or high-grade endometrial carcinoma, endometrial intraepithelial neoplasia, and mesonephric carcinoma. The other purpose was to measure the correlation between the mycotoxin levels in serum and endometrial tissue in control patients and patients with different carcinoma stages. The results would be useful for understanding the possible role of mycotoxins in endometrial tumor formation. 

## 2. Results

### 2.1. Investigated Patient Groups

Of the 25 patients involved in the study, 19 were postmenopausal, and 6 were perimenopausal. At least 23 endometrial samples were obtained in addition to the serum. In 18 cases, endometrioid endometrial carcinoma was confirmed based on the histological examination, of which 12 were low-grade and 6 were high-grade. Endometrial intraepithelial neoplasia (EIN) was confirmed in 4 more cases, and mesonephric carcinoma in 1 case.

The 27 control patients were body mass index (BMI)- and age-matched, from whom serum and endometrium samples and biometric data were collected. In this group, 21 patients were postmenopausal, and 6 were perimenopausal. In 22 cases, a sufficient amount of endometrial tissue samples was obtained; in 2 cases, the sample was sufficient for only a limited part of the examinations. One case was excluded because the histology could neither confirm nor exclude the diagnosis of EIN. In 19 cases, the histology described an atrophic endometrium or negative result. Simplex hyperplasia was confirmed in 2 cases, and endometrial polyps were confirmed in 1 case. 

During the sampling, it was fundamental that an adequate amount of histological samples was used for the necessary diagnostic tests, and only the samples obtained, in addition, were used for the mycotoxin measurements. Per test patient, 2–2.6 g endometrial tissue was used up, and for control patients, 0.5–1 g endometrial tissue was evaluated. Based on the above-described criteria, we examined four groups. The first group consisted of patients with low-grade endometrioid endometrial cancer (ECL, *n* = 12), the second group consisted of low- and high-grade endometrial cancer patients together (ECLH, *n* = 18), the third group consisted of patients with histologically confirmed EIN and simplex or complex hyperplasia (H, *n* = 6). In the control group (C), 21 patients with negative histological results were investigated.

### 2.2. Biophysical, Hormonal, Clinical-Biochemical Parameters, and Endometrium Thickness

There was no significant difference in BMI and age between the endometrial cancer and control groups. There was also no detectable difference when the control group was compared with the ECL group or separately with the high-grade group (ns. *p* > 0.2; ns. *p* > 0.3) (Table 1). Hypertension, type 2 diabetes mellitus (T2DM), and hypothyroidism were the most common comorbidities. T2DM and antidiabetic medication use were more common among patients with endometrial carcinoma (ECHL: 5/18 patient versus control group 1/21, Chi2sqare-test *p* < 0.05). There were no differences between the groups regarding AST, ALT, GGT, serum creatinine level, eGFR, TSH, serum estradiol, progesterone, and testosterone levels (Table 1). A significantly higher estrone level was found in the ECLH group compared to the control (E1: *p* < 0.05). The estradiol level showed a similar trend but did not reach a significant difference (*p* = 0.06). Regarding endometrial thickness, we also found a significant difference between EC (ECL, ECLH) patients, hyperplasia patients (H), and control patients (*p* < 0.004) (Table 1).

### 2.3. Serum and Endometrial Tissue Mycotoxin Levels

**Figure 1:**
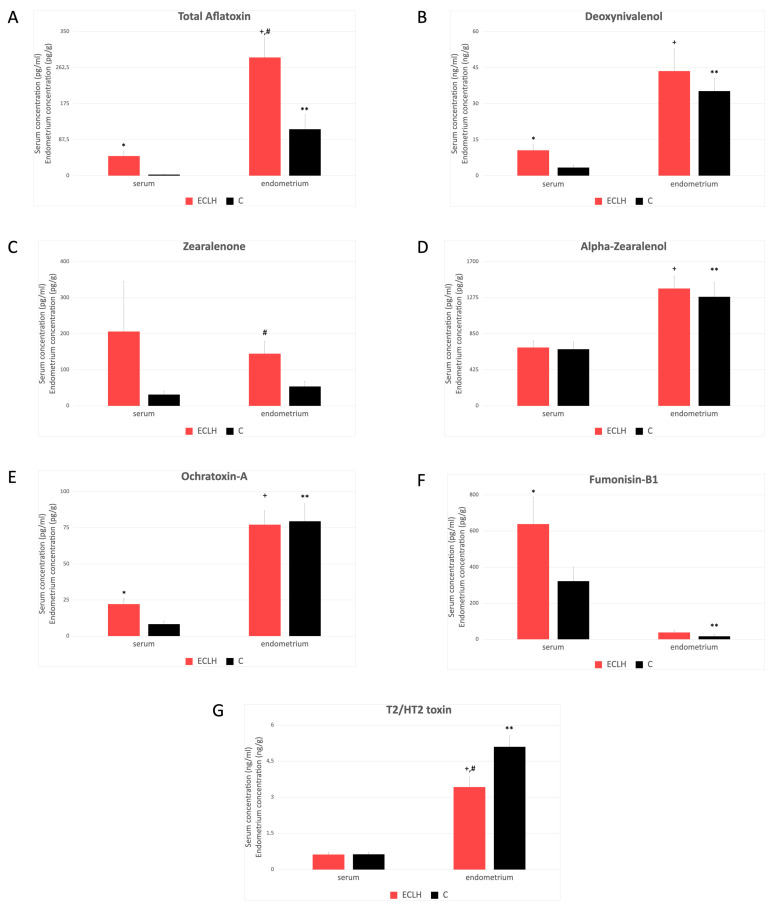
Mycotoxin level in serum and endometrial tissue (mean ± SD). ECLH: Low-grade and high-grade endometrial cancer (*n* = 18); and C: control (*n* = 21). (**A**–**G**): AFs (**A**), DON (**B**), ZEN (**C**), α-ZOL (**D**) OTA (**E**), FB1 (**F**), T2/HT2, and (**G**) concentrations in serum and endometrial tissues, measured in ECLH and C groups. We converted concentration mean values in the case of endometrium using 1.025 g/mL serum density value as a multiplier: pg/mL to pg/g, ng/mL to ng/g [17]. * ECLH serum level significantly differs from control serum level; + ECLH endometrium level significantly differs from ECLH serum level; # ECLH endometrium level significantly differs from control endometrium level; ** control endometrium level significantly differs from control serum level. [Detailed data can be found in the Supplementary Table S1] (Appendix A).

The serum mycotoxin level was significantly higher in the ECLH than in the control group in the case of total aflatoxins (AFs), deoxynivalenol (DON), ochratoxin A (OTA), and fumonisin B1 (FB1) (Figure 1A,B,E,F). However, no significant difference was found in the case of zearalenone (ZEN), α-ZOL), and T2/HT2 toxin (Figure 1C,D,G). Mycotoxin concentration of the endometrium was significantly higher in the ECLH group compared to the control in the case of AFs and ZEN but lower in the case of T2/HT2 toxin (Figure 1A,C,G). However, the mycotoxin content of the endometrium did not differ significantly between the ECLH and control group and in the case of DON, α-ZOL, OTA, and FB1 (Figure 1B,D,E,F). Comparison of the serum and endometrium mycotoxin levels was based on a conversion factor that 1 mL serum is equivalent to 1.025 g [17]. The results revealed that the mycotoxin content of the endometrium in the ECLH group contains significantly higher levels compared to the serum in of all the mycotoxins that were measured (Figure 1A,B,D,E,G), except in the case of ZEN and FB1 (Figure 1C,F). In the control group, a significantly higher mycotoxin level was found in the endometrium of all mycotoxins measured, except for FB1, which was lower in the endometrium than in the serum (Figure 1F).

**Figure 2:**
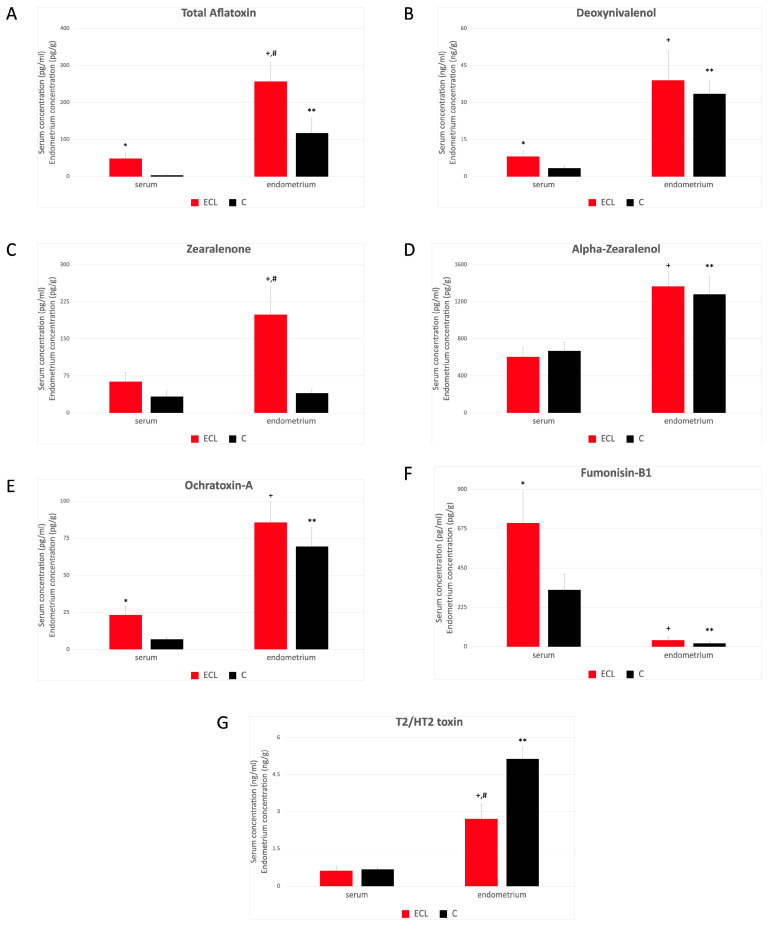
Mycotoxin level in serum and endometrial tissue (mean ± SD). ECL: Low-grade endometrial cancer (*n* = 12) and C: control (*n* = 21). (**A**–**G**): AFs (**A**), DON (**B**), ZEN (**C**), α-ZOL (**D**), OTA (**E**), FB1 (**F**), and T2/HT2 (**G**) concentrations in serum and endometrial tissues, measured in ECL and C groups. We converted concentration mean values in the case of endometrium using 1.025 g/mL of serum density value as a multiplier: pg/mL to pg/g, ng/mL to ng/g [17]. * ECL serum level significantly differs from control serum level; + ECL endometrium level significantly differs from ECL serum level; # ECL endometrium level significantly differs from control endometrium level; ** control endometrium level significantly differs from control serum level. [Detailed data can be found in the Supplementary Table S2] (Appendix A).

Mycotoxin concentrations showed different values and tendencies in the ECL group comparing the ECHL group. Elevated serum mycotoxin levels were found in the ECL group compared to the control in the case of AFs, DON, OTA, and FB1 (Figure 2A,B,E,F), but not in the case of ZEN, α-ZOL, and T2/HT2 toxin (Figure 2C,D,G). Significantly higher mycotoxin concentration was found in the endometrium in the ECL group in the case of AFs and ZEN (Figure 2A,C,G). This difference became even more obvious than the ECLH group (Figure 1A,C). On the contrary, a significantly lower mycotoxin level in the endometrium of the ECL group was found in the case of T2/HT2 toxin (Figure 2G). A comparison of the serum and endometrium levels in the ECL group showed significantly higher levels in the case of AFs, DON, α-ZOL, OTA, and T2/HT2 toxins (Figure 2A,B,D,E,G), which was the same in the case of ZEN (Figure 2C), and significantly lower in the case of FB1 (Figure 2F). A comparison of the serum and endometrium levels in the control group showed significantly higher levels in the case of AFs, DON, α-ZOL, OTA, and T2/HT2 toxins (Figure 2A,B,D,E,G), which was the same in the case of ZEN (Figure 2C). 

**Figure 3:**
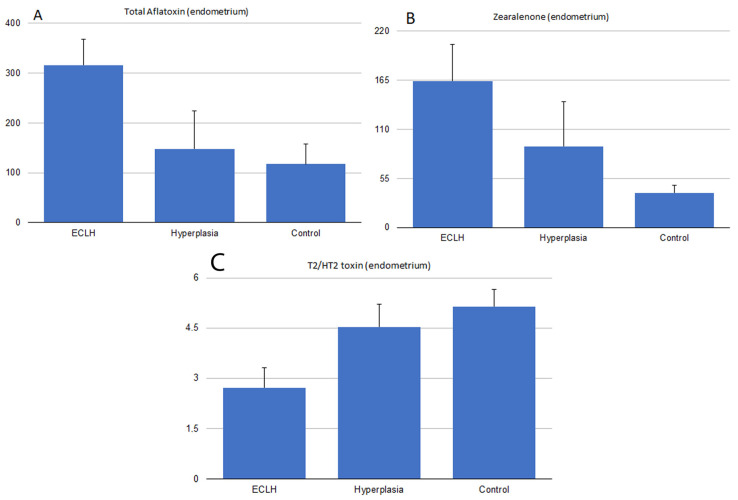
Endometrial tissue toxin concentrations in three groups (ECLH: Low-grade and high-grade, *n* = 18); (hyperplasia: simplex and complex hyperplasia and EIN, *n* = 6); (C: control, *n* = 21) measured in pg/g and ng/g. (**A**) AFs: Total aflatoxin concentration was measured in the endometrium of the three groups. All three groups significantly differ from each other. (**B**) ZEN: Zearalenon concentration was measured in the endometrium of the three groups. All three groups significantly differ from each other. (**C**) T2/HT2: Trichocetenes concentration was measured in the endometrium of the three groups. All three groups significantly differ from each other (Appendix A).

Mycotoxin concentration in the endometrium of hyperplasia (H) was also compared to the ECLH and the control groups. AF levels in the H group were between the control and the patient group levels (*p* < 0.05, Figure 3A). No differences were found in the endometrial tissue concentrations of each group for OTA (Figure 1E and Figure 2E), α-ZOL (Figure 1D and Figure 2D), DON (Figure 1B and Figure 2B), and FB1 (Figure 1F and Figure 2F). At the same time, significantly lower T2/HT2-toxin levels were measured in the ECL (*p* < 0.005) and ECLH (*p* < 0.05) groups compared to the control (Figure 1G and Figure 2G). Furthermore, compared to the H group, tissue T2/HT2-toxin concentrations were between the control and ECL group levels (*p* < 0.05, Figure 3C). 

## 3. Discussion

Patient selection was carried out over 16 weeks. The relatively short time interval was necessary because mycotoxin exposure can change from season to season and year to year according to climatic conditions [1,18]. Therefore, the comparison of samples taken in different periods and the conclusions drawn from them are questionable. However, mycotoxin exposure is unpredictable due to a lack of mycotoxin concentration in the foods consumed. The main purpose of the study was to obtain a cross-sectional picture of the differences in mycotoxin levels in blood serum and endometrial tissue among women diagnosed with endometrial cancer in menopause/perimenopause and healthy controls.

The risk factors for endometrial carcinoma are age, BMI, diabetes, and high blood pressure [19]. BMI and sudden changes in body weight may be important in the absorption of individual mycotoxins, age, and the accumulation of lipophilic molecules [20]. However, the rate of absorption of the mycotoxins is different: it is nearly complete in the case of AFs [21], and the lowest, around 4%, in the case of FB1 [22]. 

There were no differences in age or BMI between the individual groups; therefore, those were comparable. However, diabetes/insulin resistance occurred more often in the EC group in relation to the examined comorbidities, physiological functions, harmful addictions, and clinical–biochemical parameters. 

We also found a significant difference in the levels of the examined serum sexual steroids only in the case of estrone. In postmenopause, the production of sex steroids occurs primarily in fat tissue, the adrenal cortex, and endometrial tissue [23]. Estrone, estradiol (E2), and testosterone production in endometrial carcinoma tissue can be several times higher than the level detectable in normal endometrial tissue [23,24]. The background of the elevated value may be the increased expression of 17bHSD type II and the increased tissue production [25].

High-grade endometrioid endometrial cancer behaves differently from low-grade cancer in terms of prognosis, estrogen dependence, and therapy resistance. Therefore, in addition to the group including all EEC patients (ECLH), we also created another more homogeneous group containing only low-grade patients (ECL) [7]. Patients with endometrial hyperplasia (EIN, simplex hyperplasia) were placed into a separate group. Due to the relatively low number of elements in this group, only a tendency in the differences in mycotoxin concentrations was demonstrated compared to EC and C groups.

To our knowledge, this is the first study where the concentration of the above-mentioned most common mycotoxins and their metabolites were examined together in blood serum and human endometrial tissue. Our results allow us to conclude that mycotoxins accumulate in the endometrial tissue, as the tissue concentration measured in the endometrium was higher in all the investigated groups for AFs, DON, OTA, α-ZOL, and T2/HT2 toxin. This statement was also true when the examined mycotoxin level did not reach the LOQ in the serum, but it was quantifiable in the endometrium. It is known that AFB1 and AFM1 are present and accumulate in the liver [26,27], DON in the liver and kidney [28], OTA in the kidney, liver, and mammary gland tissue [29], and α-ZOL in the spleen and liver [30]. The tissue accumulation of T2/HT2 is not entirely clear, possibly due to the rapid metabolism [31,32]. Previous studies assumed that ZEN also accumulates in human endometrial tissue in endometrial cancer and hyperplastic tissues [16,33]. The results of our investigations did not support this finding. Patients of similar age were selected as controls, where endometrial atrophy was detected in most cases. Due to the lack of cyclic changes in postmenopause, the accumulation rate of some toxins may also change at the tissue level due to changing metabolic processes and hormonal and circulatory conditions; therefore, elimination may be prolonged. Due to the lack of in vivo studies, no data regarding the normal fertile endometrium are available. In the case of FB1, higher concentrations were measured in the serum than in the endometrial tissue. According to studies with different animal species, fumonisins are poorly absorbed, rapidly eliminated, and not metabolized [34].

Compared to C, significantly higher AFs, DON, and FB1 serum levels were measured in the ECLH and ECL groups. In the case of AFs, a highly significant difference was found. The mycotoxins we examined have carcinogenic and genotoxic effects even in very low concentrations [35]. The possible prognostic role of AFs and OTA in hepatocellular carcinoma (HCC) was suggested [36,37,38]. However, the results are often questionable regarding their prognostic/diagnostic value [39,40]. In the future, it would be worthwhile to conduct further studies, especially regarding the prognostic role of endometrial AF levels in EC.

The harmful effects of mycotoxins are the most obvious at the level of specific tissues and cells. The present study measured higher levels of AFs and α-ZOL in both the ECLH and ECL groups compared to the control. However, when comparing the ECL to the C group, the ZEN level was significantly higher in cancer tissue than in the controls. Other research groups reported similar results regarding endometrial ZEN concentrations [16,33,41]. In their first study [41], no ZEN was detected in normal endometrial tissue, while in the other two studies [16,33], only EC and hyperplasia tissues were examined. The difference can be explained by the fact that in the last 25 years, more sensitive detection methods and more precise preparation procedures have been invented; however, the ZEN content of foodstuffs varies by region [42]. Based on these results, it can be assumed that although AFs, ZEN, and α-ZOL are present in all endometrial tissue, these mycotoxins are more concentrated in tumor tissues. In addition, the endometrial toxin levels of the hyperplastic cases are between the levels of the cancer patient and control groups. Without drawing far-reaching conclusions, this may lead to a longer exposure and accumulation process. 

It was demonstrated that despite elimination, a continuous accumulation of ZEN was seen in an in vitro model with an MCF-7 breast cancer cell line [43]. Due to the few clinical studies available in the literature, this question is still unclear. Until now, no scientifically proven evidence exists on the tissue accumulation of AFs in endometrial tissue. However, previously published research reveals that AF accumulation could be observed in other tissues, especially tumors [44].

Regarding FB1, the present study showed that it is not accumulated in the endometrium, which would be the reason for observing no differences in the endometrial concentrations between the individual groups. No clinical data on the endometrial accumulation of DON and OTA is available, but it is assumed that they do not accumulate in tissues. Based on our present study results, they do not accumulate in the endometrium.

In the case of α-ZOL, a main metabolite of ZEN, it was found that although it accumulates in the endometrial tissue, there was no significant difference between the individual groups. At higher ZEN concentrations, an increased metabolism towards α-ZOL was observed in colon carcinoma (Caco-2) cells in vitro [39]; however, this was observed at an order of magnitude higher concentration than it was measured in our present clinical study. To clarify this statement, further gene expression studies are necessary, concentrating on the function of the enzyme 3-alpha-hydroxy dehydrogenase, which is responsible for the primary metabolism of ZEN. In contrast to the previous cases, we observed lower T2/HT2 tissue concentrations in endometrial carcinoma cases. In group H, the average values were between the average of the control and the ECHL group, which suggests that the hyperplasia patients are in an earlier phase of the process. Even though T2/HT2 accumulates in endometrial tissue, this reduced concentration did not correlate with other mycotoxin levels. Confirmation of this requires further tests with a higher number of cases. Recently, no further evidence existed to explain these results in the literature. 

Mycotoxins, especially aflatoxins, can exert carcinogenic effects through various mechanisms. One of the most toxic and carcinogenic metabolites is 8,9-dihydro-(8-N7-guanyl)-9-hydroxy-AFB1 [45]. In addition to direct DNA damage, overloading of proliferation cascades, and immunosuppression, it can inhibit the function of DNA repair genes PARP1 and MLH1 [46,47]. The role of TP53 in carcinogenic AFB1 metabolite-induced mutations has been confirmed [48]. One study showed that in response to DNA damage induced by sterigmatocystin (an aflatoxin precursor), hMLH1 was first activated, followed by ERK, p38, and p53 activation, and finally resulted in G2 arrest in an oesophageal squamous cell line [47]. Increased expression of similar signal transduction pathways was also described in the case of OTA [49].

Classification based on molecular differences is part of the pathological–histological diagnosis of EEC. Performing TP53 mutation, MMR deficiency (MMRd), and, in necessary cases, POLE tests is a daily practice. Thus, we know some molecular differences based on the histological results. TP53 mutation occurs relatively rarely in about 15% of EEC cases. MMRd cases may be present in up to 30% of cases [7]. In our study, 5 MMRd cases out of 18 were registered. Compared with endometrial Afs levels, we found a correlation between MMRd cases and aflatoxin levels (r = 0.50, *p* < 0.05, 95%CI: 0.08425 to 0.8220). However, no significant relationship was found for the other mycotoxins.

## 4. Conclusions

In the present study, the co-presence and levels of the most frequently occurring carcinogenic and cytotoxic mycotoxins in human endometrial tissue in endometrial cancer patients were investigated for the first time. The results revealed that the tested mycotoxin levels in the serum differed from those in endometrial tissue and that mycotoxins accumulated in the endometrial tissue. Elevated AFs, DON, and FB1 serum levels and higher endometrial concentrations of AFs and ZEN were detected compared to the control. EC patients have the same level as controls in the case of FB1 and a lower T2/HT2 level in the endometrial tissue. We found a correlation between MMRd and AFs endometrial levels.

## 5. Materials and Methods

### 5.1. Patients and Sample Collection

Serum and endometrial tissue samples were collected between 8 September 2023 and 14 December 2023 from patients either treated for a previously histologically confirmed endometrial cancer or reasonable suspicion. We also took samples from control patients who underwent treatment/surgery for other non-malignant reasons in the same period. All patients received detailed information, and sampling took place based on the prior permission of the regional ethics committee after signing the relevant consent form. In all cases, the blood and tissue samples (2–2.6 g endometrial tissue from patients with endometrial cancer and 0.5–1 g endometrial tissue from control patients) were taken after fasting for at least 12 h. In addition to the sampling, we collected data on weight, age, obstetric history, comorbidities, possible medication, smoking, and alcohol consumption, evaluated the patients’ liver and kidney function laboratory values, and measured the thickness of the endometrium during an ultrasound examination. We excluded patients who had malabsorption, severe kidney or liver failure, thyroid disease, or who had a significant change in weight in the past period. 

### 5.2. Experimental Setup on Samples

Afs (B1, B2, G1, G2), DON, FB1, OTA, ZEN, α-ZOL, T2/HT2 toxins, estradiol, progesterone, estrone, and testosterone levels were measured from the serum samples taken according to the methodology detailed below. All endometrial samples taken were histologically evaluated. In the cases of endometrial cancer, a mutation analysis, essential for diagnosis, was also conducted. The seven mycotoxins mentioned above were measured from the endometrial and serum samples.

### 5.3. Mycotoxin Analyses

Mycotoxin measurements were performed on endometrial tissue and serum samples from each group. The assays were performed using ELISA method optimization for the serum and organ/tissue samples [50].

ZEN, OTA, FB1, DON, T2/HT2 toxin, and Afs (total aflatoxins, B1, B2, G1, and G2) were analyzed using immunoassays. In the case of α-ZOL, a GC-MS measurement was applied.

After thawing, the stored endometrium samples were washed with sterile 1X concentrated PBS to remove blood. After this process, the samples were ground and homogenized with ceramic balls. Aliquots were weighed into extraction tubes and stored at −70 °C until further analysis.

#### 5.3.1. Zearalenone Assay

ZEN analyses used commercial Ridascreen Zearalenone (Art No.: R1401 R-Biopharm, Arnhem, Germany) enzyme immunoassay kits. For the serum sample preparation RIDA© C18 column (art No: R2002, R-Biopharm, Arnhem, Germany) was used according to the manufacturer’s instructions, and samples were measured in triplicates. Endometrial tissue was homogenized with FastPrep-24 Classic (MP Biomedicals, Irvine, CA, USA) homogenizer in ice-cold sodium acetate buffer of 50 mM (pH = 4.8) and incubated for 3 h at 37 °C in a shaker with the addition of *Helix pomatia* β-glucuronidase/aryl sulfatase (BGALA-RO, Roche, Basel, Switzerland) according to the manufacturer’s instructions. Extraction was made with a mixture of 70% (*v*/*v*) methanol and water (3:1 *v*/*v*). The extracts were centrifuged at room temperature (RT) at 8000× *g* for 5 min, and the supernatants were collected and diluted with assay buffer. Measurements were acquisited and data were analyzed with a microplate reader Thermo Multiskan^TM^ FC (Waltham, MA, USA) equipped with the SkanIt RE software (version 6.1.1.7). The absorbance was measured at 450 nm with a 630 nm reference wavelength.

#### 5.3.2. α-Zearalenol

The blood serum sample preparation for α-zearalenol was the same as for ZEN.

ZEN metabolite: α-ZOL in blood serum and endometrial tissue were tested in triplicate by gas chromatograph with a mass spectrometer method (GC-MS) (Finnigan Trace/DSQ, Thermo Electron Corp., Austin, TX, USA) equipped with a 30 m capillary column (Rxi 5 ms, 0.25 mm ID, 0.25 micron df, Restek, Bellefonte, PA, USA).

The blood serum sample preparation for α-zearalenol was the same as for ZEN. Tissue samples were homogenized in ice-cold 50 mM sodium acetate buffer (pH 4.80) by a FastPrep-24 classic homogenizer and then incubated for 3 h at 37 °C with the addition of *Helix pomatia* β-glucuronidase/aryl sulfatase. Extraction was performed with a mixture of 70% (*v*/*v*) methanol and water (3:1 *v*/*v*). The extracts were centrifuged at RT at 8000× *g* for 5 min and purified using a RIDA C18 column. After elution, the extract was evaporated and reconstructed with methanol/ACN (80:20 *v*/*v*%). In total, 1 µL of sample volumes were injected at a 30 mxmin^−1^ split ratio using the hot-needle technique. The injection temperature was 230 °C, and the interface and ion source temperatures were set to 250 °C. Helium was the carrier gas at a constant flow rate of 1 mlxmin^−1^. The temperature program was 12.5 min isothermal heating at 90 °C for 2 min, followed by an oven temperature ramp of 5 °C per minute to 330 °C. The system was temperature-equilibrated for 2 min at 90 °C before injecting the next sample [51].

#### 5.3.3. Ochratoxin-A

Serum samples were melted and diluted/extracted with a threefold ACN/water solution (84/16, *v*/*v*) and shaken in an orbital shaker for 15 min at RT. Extracts were centrifuged at RT at 8.000× *g* for 5 min; the supernatants were collected and diluted with 0.01 M PBS, pH 7.4. Endometrium tissue samples were homogenized in ice-cold 50 mM sodium acetate buffer (pH = 4.80) with a bench-top bead-beating lysis system. Homogenates were then incubated for 3 h at 37 °C by adding β-glucuronidase/arylsulfatase from *Helix pomatia* [46]. Following incubation, the homogenized sample was diluted with an ACN/water solution (84/16, *v*/*v*) and shaken (extracted) for 15 min. Next, the extracts were centrifuged at 10.000× *g* for 10 min at 4 °C. The supernatant was diluted 100× with 0.01 M phosphate-buffered saline (PBS, pH = 7.40), and the diluted extracts were used for measurements in the assay. Determination of OTA was performed with an ELISA kit (TOXI-WATCH Ochratoxin A ELISA Kit, Cat. Nr.: 3000051, Soft Flow Ltd., Pécs, Hungary) according to the manufacturer’s instructions and was measured in triplicates. The recovery was 68.9–90.6%, measured with the OTA-spiked endometrial samples.

#### 5.3.4. Total Aflatoxin and Deoxynivalenol

The AFs and DON contents were determined using the Toxi-Watch (Soft Flow Ltd., Pécs, Hungary) ELISA kits, which were previously validated for serum samples and different organs/tissues according to the manufacturer’s instructions. Serum samples were melted and diluted/extracted with a threefold EOH/water solution (23/77, *v*/*v*) and shaken in an orbital shaker for 15 min at RT. Extracts were centrifuged at RT at 8.000× *g* for 5 min; the supernatants were collected and, in the case of DON, diluted 10 times with 0.01 M PBS, pH 7.4. Samples were measured in triplicates. The recovery was 74.3–97.4% for aflatoxin B1-spiked endometrial tissue samples and 74.56–94.56% for DON, respectively.

#### 5.3.5. Fumonisin B1

FB1 was measured using EUROPROXIMA Fumonisin (5121FUM) (R-Biopharm, Arnhem, Germany) assay kit. This commercial kit was validated for serum and different animal tissues. Before the analyses of the endometrial samples, we tested the recovery rate of FB1 spiked tissue, which was 69.23–78.76%. The analyses were performed according to the manufacturer’s instructions. Samples were measured in triplicates.

#### 5.3.6. T2/HT2-Toxin Analyses

Bio-Shield T2/HT-2 (Prognosis Biotech, Larissa, Greek) ELISA kits were used to determine T2/HT2 toxins according to the manufacturer’s instructions. The blood serum sample preparation for the T2/HT-2 toxin was the same as that for ZEN. Samples were measured in triplicates. Following evaporation, the dried residue was redissolved in 500 µL of methanol–water (35:65 *v*/*v*%). By endometrial samples, the tissue was extracted with acetonitrile (ACN) after evaporation; the dried residue was redissolved in 500 µL of methanol–water (35:65 *v*/*v*%).

### 5.4. Hormone Analyses

For the quantitative determination of estrone (E1) in serum, ALPCO Estrone ELISA kits were used (Cat. No. 11-ESRHU-E01; Salem, MA, USA). The immunoassay was performed according to the manufacturer’s instructions, and serum samples were measured in triplicates.

For steroid analyses in serum, a 17-beta-estradiol (Cat No: DNOV003, NovaTec Immundiagnostica, Dietzenbach, Germany), a Progesterone (Cat No: DNOV006, NovaTec, Dietzenbach, Germany), and a Testosterone (Cat No: DNOV002, Novatec, Dietzenbach, Germany) kit were used. The immunoassay was performed according to the manufacturer’s instructions, and serum samples were measured in triplicates.

### 5.5. Statistical Analysis

The statistical analyses were carried out using the GraphPad Prism software (Version 10.1.1. 2023, GraphPad, La Jolla, CA, USA). Continuous variables were compared with an independent t-test or Mann–Whitney test. Comparisons between groups were performed using a one-way ANOVA test. Linear regression was used to analyze independent correlated factors; Pearson correlation was used to determine the relationship between values, and multiple logistic regression was used to predict the influence of different variables. *p*  <  0.05 was considered to indicate statistical significance.

## Figures and Tables

**Table 1:** Biophysical, hormonal, clinical-biochemical parameters, and endometrium thickness of the study groups (mean ± SD).

	ECL (*n* = 12)	ECLH (*n* = 18)	Control (*n* = 21)
BMI (Mean ± SEM, kg/m^2^)	34.25 ± 2.141	33.97 ± 1.701	30.43 ± 1.415
ALT (GPT, Mean ± SEM, IU/L)	19.45 ± 2.977	18.36 ± 2.486	25.07 ± 2.958
TSH (Mean ± SEM, mIU/L)	2.139 ± 0.7526	2.21 ± 0.5885	1.33 ± 0.5
Progesterone (Mean ± SEM, ng/mL)	0.27 ± 0.0932	0.41 ± 0.1639	0.41 ± 0.1544
Estrone (Mean ± SEM, pg/mL)	45.30 ± 6.075 ^ab^	59.12 ± 13.9 ^b,^*	27.90 ± 6.209 ^a^

^a,b^ Different superscripts of the same line mean significant difference at *p* ˂ 0.05 level. *—*p* < 0.05; **—*p* < 0.001.

## Data Availability

The raw data supporting the conclusions of this manuscript will be made available by the authors, without undue reservation, to any qualified researcher.

## Supplementary Materials

The following supporting information can be downloaded at: https://www.mdpi.com/article/10.3390/toxins16060236/s1, Table S1. (data for Figure 1): Mycotoxin level in serum and endometrial tissue (mean±SD). C: control; ECLH: Low-grade and High-grade endometrial cancer. Table S2. (data for Figure 2): Mycotoxin level in serum and endometrial tissue (mean±SD). ECL: Low-grade endometrial cancer and C: control.

## Appendix A

Figure 1: (Figure 1A) AFs (ECLH serum 47.67 ± 11.75 pg/mL; endometrium 287 ± 48.32 pg/g and C serum 2.875 ± 1.308 pg/mL; endometrium 113 ± 35.4 pg/g; ECLH P < 0.0001 and C P < 0.005). (Figure 1B) DON (ECLH serum 10.65 ± 2.391 ng/mL; endometrium 43.58 ± 9.306 ng/g and C serum 3.417 ± 0.9871 ng/mL; endometrium 35.25 ± 5.423 ng/g; ECLH P < 0.005; C P < 0.0001). (Figure 1C) ZEN (ECLH 243.2 ± 167.7 serum pg/mL; endometrium 164± 40.75 pg/g and C serum 33.46 ± 11.22 pg/mL; endometrium 45.01 ± 10.67 pg/g; ECLH P < 0.001; C P < 0.01). (Figure 1D) A-ZOL (ECLH serum 688 ± 83.94 pg/mL; endometrium 1383 ± 146.3 pg/g and C serum 668.4 ± 90.33 pg/mL; endometrium 1286 ± 169.9 pg/g; ECLH P < 0.001; C P < 0.01). (Figure 1E) OTA (ECLH serum 22.1 ± 4.132 pg/mL; endometrium 77.09± 9.841 pg/g and C serum 8.25 ± 2.09 pg/mL; endometrium 79.45 ± 12.27 pg/g; ECLH and C P < 0.001). (Figure 1F) FB1 (ECLH serum 638.1 ± 148.7 pg/mL; endometrium 37.77 ± 12.65 pg/g and C serum 322.5 ± 79.9 pg/mL; endometrium 17.1 ± 10.37 pg/g; ECLH and C P < 0.001, separately). (Figure 1G) T2/HT2 toxins (ECLH serum 0.6188 ± 0.1017 ng/mL; endometrium 3.425 ± 0.419 ng/g and C serum 0.6257 ± 0.09578 ng/mL; endometrium 5.099 ± 0.4555 ng/g; ECLH and C P < 0.0001, respectively)

Figure 2: (Figure 2A) AFs (ECL serum 48.79 ± 15.38 pg/mL; endometrium 257 ± 51.09 pg/g and C serum 3.286 ± 1.477 pg/mL; endometrium 117.3 ± 40.56 pg/g; ECL and C P < 0.001). (Figure 2B) DON (ECL serum 8.123 ± 2.293 ng/mL; endometrium 39.04 ± 11.91 ng/g and C serum 3.417 ± 0.9871 ng/mL; endometrium 33.6 ± 5.417 ng/g; ECL P < 0.005; C P < 0.0001). (Figure 2C) ZEN (ECL serum 63.16 ± 19.25 pg/mL; endometrium 198.9 ± 56.61 pg/g and C serum 33.46 ± 11.22 pg/mL; endometrium 39.71 ± 8.238 pg/g; ECL P < 0.001; C P < 0.01). (Figure 2D) A-ZOL (ECL serum 606.1 ± 107.5 pg/mL; endometrium 1366 ± 158.4 pg/g and C serum 668.4 ± 90.33 pg/mL; endometrium 1280 ± 194.6 pg/g; ECL P < 0.001; C P < 0.01). (Figure 2E) OTA (ECL serum 23.32 ± 5.894 pg/mL; endometrium 85.67 ± 13.72 pg/g and C serum 6.762 ± 1.546 pg/mL; endometrium 69.47 ± 12.67 pg/g; ECL and C P < 0.001). (Figure 2F) FB1 (ECL serum 707.5 ± 184.5 pg/mL; endometrium 37.5 ± 18.66 pg/g and C serum 325.2 ± 89.83 pg/mL; endometrium 19.94 ± 12.01 pg/g; ECL and C P < 0.001, separately). (Figure 2G) T2/HT2 toxins (ECL serum 0.6122 ± 0.1637 ng/mL; endometrium 2.714 ± 0.5955 ng/g and C serum 0.6655 ± 0.09851 ng/mL; endometrium 5.141 ± 0.5133 ng/g; ECL and C P < 0.0001, respectively)

Figure 3: (Figure 3A) AFs: Total Aflatoxin concentration was measured in the endometrium of the three groups. All three groups significantly differ from each other: ECLH endometrium 316 ± 52.15 pg/g; H endometrium 147.8 ± 76.05 pg/g; C endometrium 117.3 ± 40.56 pg/g (*p* < 0.05, OW-ANOVA); (Figure 3B) ZEN: Zearalenone concentration measured in the endometrium in the three groups. All three groups significantly differ from each other: ECLH endometrium 164 ± 40.75 pg/g; H endometrium 90.8 ± 49.66 pg/g; C endometrium 38.85 ± 8.155 pg/g (*p* < 0.05, OW-ANOVA); (Figure 3C) T2/HT2: mycotoxin concentration measured in the endometrium in the three groups. All three groups significantly differ from each other: ECLH endometrium 2.714 ± 0.5955 pg/g; H endometrium 4.533 ± 0.6871 pg/g; C endometrium 5.141 ± 0.5133 pg/g (*p* < 0.05, OW-ANOVA)
