# Supplementary material for: The Possible Role of Mycotoxins in the Pathogenesis of Endometrial Cancer
Source: Toxins (Basel). 2024 May 23;16(6):236. doi: 10.3390/toxins16060236 (PMC11209310; doi:10.3390/toxins16060236)
Supplement: Supplementary file 1 [file toxins-16-00236-s001.zip › toxins-2965006-supplementary.pdf]

toxins

MDPI

Supplementary Material

The possible role of mycotoxins in the pathogenesis of endometrial cancer

Márkó Unicsovics, Zsófia Molnár, Miklós Mézes, Katalin Posta, György Nagyéri, Szabolcs Várbíró, Nándor Ács, Levente Sára and Zsuzsanna Szőke

Table S1. Mycotoxin level in serum and endometrial tissue (mean±SD). C: control; ECLH: Low-grade and High-grade endometrial cancer.

Toxin	Sample type	Group	sample size	Min.	Max.	Mean±SEM
Total aflatoxin	Serum conc. (pg/ml)	ECLH	20	0	208	47.67±11.75*
		C	21	0	24	2.875±1.31
	Endometrium conc. (pg/g)	ECLH	18	0	735	287.0±48.32$^{+,\#}$
		C	18	0	672	113.0±35.4**
Deoxynivalenol	Serum conc. (ng/ml)	ECLH	21	0	47.03	10.65±2.39*
		C	24	0	17.88	3.417±0.99
	Endometrium conc. (ng/g)	ECLH	18	0.5	139.1	43.58±9.30^{+}
		C	21	0.5	93.18	35.25±5.42**
Zearalenone	Serum conc. (pg/ml)	ECLH	20	0	3404	243.2±167.7
		C	21	0	241.2	33.46±11.22
	Endometrium conc. (pg/g)	ECLH	18	0	686.5	164± 40.75$^{\#}$
		C	18	0	134.9	45.01± 10.67
Alpha-Zearalenol	Serum conc. (pg/ml)	ECLH	20	23	1712	688±83.94
		C	21	106	1904	668.4±90.33
	Endometrium conc. (pg/g)	ECLH	18	321	2841	1383± 146.3^{+}
		C	21	215	3624	1286± 169.9**
Ochratoxin-A	Serum conc. (pg/ml)	ECLH	20	0	84	22.1±4.132*
		C	21	0	24	8.25± 2.09
	Endometrium conc. (pg/g)	ECLH	18	16	168	77.09± 9.84^{+}
		C	19	8	240	79.45± 12.27**
Fumonisin-B1	Serum conc. (pg/ml)	ECLH	18	0	2640	638.1± 148.7*
		C	21	0	1610	322.5±79.9
	Endometrium conc. (pg/g)	ECLH	18	0	212	37.77±12.65
		C	21	0	218	17.1± 10.37**
T2/HT2 toxin	Serum conc. (ng/ml)	ECLH	20	0	1.6	0.619±0.102
		C	21	0	1.572	0.622±0.095
	Endometrium conc. (ng/g)	ECLH	18	0.15	7.92	3.425±0.419$^{+,\#}$
		C	17	1.504	9.32	5.099± 0.455**

Table S1 (for Figure 1.) Mycotoxin concentrations in serum and endometrial tissues, measured in ECL and C groups. We converted concentration mean values in the case of endometrium using 1,025g/ml se-rum density value as a multiplier: pg/ml to pg/g, ng/ml to ng/g.* ECLH serum level significantly differs from control serum level; + ECLH endometrium level significantly differs from ECLH serum level; # ECLH endometrium level significantly differs from control endometrium level; ** Control endometrium level significantly differs from control serum level.

Table S2. Mycotoxin level in serum and endometrial tissue (mean±SD). ECL: Low-grade endometrial cancer and C: control.

Toxin	Sample type	Group	sample size	Min.	Max.	Mean±SEM.
Total aflatoxin	Serum conc. (pg/ml)	ECL	14	0	208	48.79±15.38*
		C	21	0	24	3.29 ±1.48
	Endometrium conc. (pg/g)	ECL	12	0	528	257.0±51.09$^{+,\#}$
		C	18	0	672	117.3±40.56**
Deoxynivalenol	Serum conc. (ng/ml)	ECL	14	0	27.53	8.123±2.293*
		C	24	0	17.88	3.417±0.987
	Endometrium conc. (ng/g)	ECL	12	0.5	139.1	39.04±11.91^{+}
		C	21	0.5	93.18	33.6±5.42**
Zearalenone	Serum conc. (pg/ml)	ECL	14	0	289.4	63.16±19.25
		C	21	0	241.2	33.46±11.22
	Endometrium conc. (pg/g)	ECL	12	0	686.5	198.9± 56.61$^{+,\#}$
		C	19	0	103.1	39.71± 8.24
Alpha-Zearalenol	Serum conc. (pg/ml)	ECL	14	286	1712	606.1±107.5
		C	21	106	1904	668.4±90.33
	Endometrium conc. (pg/g)	ECL	12	652	2320	1366± 158.4^{+}
		C	19	215	3624	1280± 194.6**
Ochratoxin-A	Serum conc. (pg/ml)	ECL	14	0	68	23.32±5.894*
		C	21	0	24	6.762±1.546
	Endometrium conc. (pg/g)	ECL	12	16	168	85.67±13.72^{+}
		C	19	8	240	69.47± 12.67**
Fumonisin-B1	Serum conc. (pg/ml)	ECL	12	0	1950	707.5±184.5*
		C	21	0	1610	325.2±89.83
	Endometrium conc. (pg/g)	ECL	12	0	212	37.5±18.66^{+}
		C	18	0	218	19.94±12.01**
T2/HT2 toxin	Serum conc. (ng/ml)	ECL	14	0	1.6	0.612±0.164
		C	21	0	1.572	0.665±0.098
	Endometrium conc. (ng/g)	ECL	12	0.15	7.92	2.714±0.595$^{+,\#}$
		C	17	1.504	9.32	5.141± 0.51**

Table S2 (for Figure 2.) Mycotoxin concentrations in serum and endometrial tissues, measured in ECL and C groups. We converted concentration mean values in the case of endometrium using 1,025g/ml se-rum density value as a multiplier: pg/ml to pg/g, ng/ml to ng/g.* ECL serum level significantly differs from control serum level; + ECL endometrium level significantly differs from ECL serum level; # ECL endometrium level significantly differs from control endometrium level; ** Control endometrium level significantly differs from control serum level.
